# The effect of diabetes on corneal endothelium: a meta-analysis

**DOI:** 10.1186/s12886-020-01785-3

**Published:** 2021-02-10

**Authors:** Kaikai Zhang, Liangliang Zhao, Chao Zhu, Weijin Nan, Xinfen Ding, Yuchen Dong, Meisheng Zhao

**Affiliations:** Department of Ophthalmology, The Second Affiliated Hospital of Jilin University, Changchun, 13000 China

**Keywords:** Diabetes mellitus, Corneal endothelium, Meta-analysis

## Abstract

**Background:**

This research was conducted with the aim to determine the effect of diabetes mellitus on corneal endothelial cells.

**Methods:**

The terms: (“diabetes mellitus” or “diabetes” or “diabetic”) and (“corneal endothelium” or “cornea” or “Corneas”) searched in Pubmed, Embase, Cochrane, and Web of science until August 2019. The included types of studies contained observational studies. The standard mean difference (SMD) which was deemed as main size effects for continuous data was calculated by means and standard deviations. The data on corneal endothelial cell density (ECD), mean cell area (MCA), cell area variation coefficient (CV) and percentage of hexagonal cells (HEX) included in the study were collected and analyzed using stata15.1.

**Results:**

The final 16 cross-sectional studies and 2 case-control studies were included for the meta-analysis. Meta-analysis revealed that diabetes mellitus could reduce ECD (SMD = − 0.352, 95% CI -0.538, − 0.166) and the HEX (SMD = − 0.145, 95% CI -0.217, − 0.074), in addition to increasing CV (SMD = 0.195, 95% CI 0.123, 0.268). Nevertheless, there was no statistically significant differences observed when combining MCA (SMD = 0.078, 95% CI -0.022, 0.178). In subgroup analysis, Type 2 diabetes patients owned less corneal ECD (*P* < 0.05). Moreover the same results also found during the subgroup form Asia, Europe and American. The meta-regression revealed the type of diabetes mellitus might be contributing to heterogeneity. (*P* = 0.008). The results indicated a significant publication bias for studies, with combined CV (Begg’s test, *P* = 0.006; Egger’s test, *P* = 0.005) and merged combined HEX (Begg’s test, *P* = 0.113; Egger’s test, *P* = 0.024).

**Conclusions:**

As indicated by meta-analysis, diabetes mellitus could cause a detrimental effect on corneal endothelium health. Diabetes mellitus contributed to the instability of corneal endothelium during the analysis. Therefore, further research is considered necessary to confirm our research results.

**Trial registration:**

CED 42019145858.

**Supplementary Information:**

The online version contains supplementary material available at 10.1186/s12886-020-01785-3.

## Background

Corneal endothelium refers to a layer of hexagonal cells located on the posterior surface of the cornea and is responsible for maintaining corneal transparency through barrier and pump functions [1–3]. After birth, the number of corneal endothelial cells will decline [4], and age is known as a significant cause of endothelial cell loss, the rate of which could reach 0.6% annually [5]. In addition, trauma [6, 7], contact lens [8], infection [9], ultraviolet light [10], smoking [11], intraocular surgery [12, 13] can also lead to endothelial cell loss. Kudva’s study found that diabetes patients lost an average of 27.5% endothelial cells at 3 months after surgery, but not diabetes patients lost 18.3% [14]. Moreover, Diabetes may become a risk factors the failure of donor tissue for DMEK [15]. The good health of corneal endothelium is regarded as a guarantee for postoperative recovery. However, the diabetic cornea presents a high level of risk. Intraocular surgery results in the extension of corneal edema and incision healing time, and in some cases, can even cause corneal endothelium to be decompensated [16]. As the corneal endothelium lacks the capability of regeneration, the loss of corneal endothelium will be compensated for by the expansion and migration of neighboring cells. The damage caused to endothelial cells can lead to corneal stromal hydration and vision loss, which can only be reversed by corneal transplantation [17]. However, this could impose a heavy burden on patients both financially and psychologically.

Diabetes is a universal health problem with a prevalence ranging from 15.2 to 42.4% [18] and is estimated to be among the seven leading causes of death by 2030 [19]. The detrimental effects of diabetes on the eyes include diabetic retinopathy [20], cataract [21], glaucoma [22, 23], and corneal diseases [24]. As for the cornea, the negative effects include corneal epithelial lesions [25], increased corneal thickness [26], while the extent of impact on the corneal endothelium differs among previous studies. In order to attract the attention of doctor, the corneal endothelium is protected during the operation. Therefore, the meta-analysis was conducted to investigate the density of corneal endothelial cells (ECD), mean cell area (MCA), cell area variation coefficient (CV), and the percentage of hexagonal cells (HEX) in diabetes. In the meantime, healthy people were also involved in assessing the effects of diabetes on the corneal endothelium.

## Method

This meta-analysis was carried out in accordance with the Preferred Reporting Items for Systematic Reviews and Meta-analyses (PRISMA) statement checklist [27].

### Search strategies and research options

The original documentations were retrieved from Medline, Cochrane, EMBASE, and Web of Science databases as of August 1, 2019. Use terms: (“diabetes mellitus” or “diabetes” or “diabetic”) and (“corneal endothelium” or “cornea” or “Corneas”) for topic or mesh search. Two investigators (Zhang KK and Zhao LL) were deployed to complete the preliminary screening of abstracts and titles independently. Then, each potential full-text study was screened in line with the prescribed inclusion criteria, and the references for included literature were manually retrieved to find the literature with potential relevance. Any disagreement was resolved by discussion and consultation with a third author (Zhu C).

For the study, the inclusion criteria were set as follows: 1. Corneal endothelial studies in diabetes and healthy people; 2.Observational studies published as original literature; 3. English literature published as of August 1, 2019; 4. Exclusion Intraocular surgery, eye diseases, incomplete literature data, wearing contact lenses, in vitro tests, trauma, case report, meeting, letter, and review.

#### Data extraction and evaluation of the quality

In order to minimize publication bias, two independent authors (Zhang KK and Zhao LL) were deployed to extract the data based on a standardized collection. The collected information includes the first author’s name, the date of publication, race, country, age, gender, study design, measurement tool, type of diabetes mellitus, sample size, corneal endothelial cell density (ECD), mean cell area (MCA), cell area variation coefficient (CV). Mean value and standard deviation were applied to calculate the Standard Mean Difference (SMD). To evaluate the quality of the induced study, The Newcastle-Ottawa scale (NOS) was employed to assess the quality of the selected studies [28]. Any disputes were resolved by discussion to reach a consensus with a third author (Zhu C).

#### Statistical analysis

Stata15.1 software was applied to perform all of the statistical analyses. Means and standard deviations of continuous outcomes were used to perform the calculation of the Standard Mean Difference. A fixed-effects model was applied in case of no apparent heterogeneity(I^2^<50%)for the minimum deviation [29]. Otherwise, a Table.1 random-effects model was applied. Subgroup analyses and Meta-regression were conducted to identify the source of heterogeneity. Moreover, a sensitivity analysis was carried out to determine whether some original studies could be contributory to heterogeneity. Egger’s test and Begg’s test were conducted to evaluate the potential publication bias [45, 46]. The *P* < 0.05 was treated as statistically significant.

**Table 1:** summaries the characteristics and NOS score of the selected studies

Authors	Year	Race	Country	Sample size		Gender(M/F)		Age		Study design	Diabetes mellitus type	Measure tool	NOS
				Cases	Controls	Cases	Controls	Cases	Controls				
Keoliean [30]	1992	Caucasians	America	14	14	Nm	Nm	33 ± 12	33 ± 10	CS	Type 1	Widefieldspecular microscope	6
Larsson [31]	1996	Caucasians	America	49	20	Nm	Nm	36 ± 12	36 ± 12	CS	Type 1	Broomall Pa	6
				60	20	Nm	Nm	60 ± 10	59 ± 12	CS	Type 2	Broomall Pa	6
Siribunkum [32]	2001	Asians	Thailand	60	60	20/10	20/10	60.0 ± 9.1	60.4 ± 11.7	CC	Type 1 and 2	EM-1020	5
Inoue [33]	2002	Asians	Japan	99	97	53/46	52/45	65.5 ± 7.5	67.6 ± 7.3	CS	Type 2	NONCON ROBO CA	6
Cho o[34]	2010	Asians	Malaysia	100	100	Nm	Nm	Nm	Nm	CC	Type 2	SP-3000P	7
Jr [35]	2010	Caucasians	Hungary	41	40	12/9	13/9	40.97 ± 15.46	40.45 ± 15.16	CS	Type 1	EM-1000	7
				59	60	10/20	15/15	64.36 ± 10.47	62.69 ± 13.38	CS	Type 2	EM-1000	7
Sudhir [36]	2012	Asians	India	1191	120	637/554	58/62	54.8 ± 9.5	51.9 ± 8.7	CS	Type 2	SP-8000	6
Paulsen [37]	2013	Caucasians	Denmark	107	128	51/56	49/79	72.1 ± 11.0	75.6 ± 8.9	CS	Type 2	SP-2000P	6
Urban [38]	2013	Caucasians	Poland	123	124	60/63	66/58	15.34 ± 3.06	14.58 ± 2.01	CS	Type 1	SP-2000P	6
Arici [39]	2014	Caucasians	Turkey	60	109	12/18	18/33	54.8 ± 9.6	53.3 ± 8.2	CS	Type 2	SP-3000P	6
Wichi [40]	2015	Asians	Thailand	171	156	41/49	34/56	58.49 ± 9.78	58.98 ± 13.12	CS	Type 1 and 2	CS 4	4
Galgauskas [41]	2015	Caucasians	Lithuania	123	120	31/31	32/33	45.5 ± 13.4	45.4 ± 19.5.	CS	Type 2	SP-9000	7
Wichai [42]	2016	Asians	Thailand	271	82	63/85	16/30	61.04 ± 9.51	57.93 ± 11.30	CS	Type 1 and 2	CS 4	5
Islam [43]	2017	Asians	Pakistan	149	149	89/60	77/72	54.13 ± 9.97	52.01 ± 12.10	CS	Type 1 and 2	SP-3000P	7
Amira [44]	2017	Caucasians	Saudi	57	45	27/30	22/23	57.08 ± 8.37	50.80 ± 8.39	CS	Type 2	EM-3000	5
Veysel [11]	2018	Caucasians	Turkey	153	146	76/77	71/75	54.9 ± 6.6	53.9 ± 7.3	CS	Type 2	SP-3000P	7

*CS* Cross-sectional, *CC* Case-control, *NM* Not Mention

## Results

**Fig. 1:**
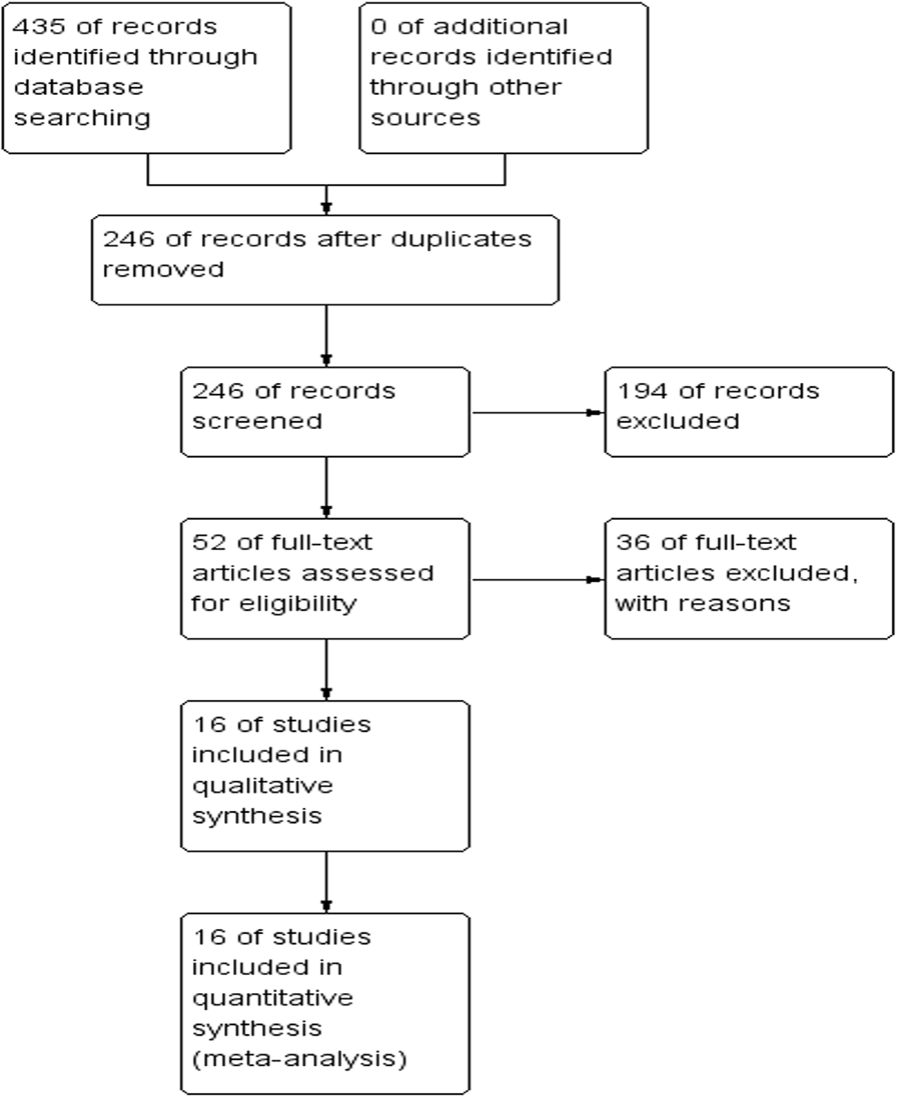
The PRISMA diagram for systematic search and screening process

The entire process of study screening is illustrated in Fig. 1. A total of 435 articles were identified using the four academic databases, of which 189 articles were assessed after all duplicate ones were removed. Besides, 194 articles were excluded due to titles and abstracts. The remaining 52 full-text articles were then assessed for their eligibility. Finally, 16 articles [11, 30–44] including 16 cross-sectional studies and 2 case-control studies that could meet the inclusion criteria were included in our meta-analysis through a review of the full text, of which 36 were excluded as well because 2 articles were not written in English, 10 articles were of incomplete text, 4 articles contained the data that could not be extracted, and 20 articles were related to animal experiments. Therefore, the existing 4470 samples included in our meta-analysis involving 2887 cases and 1583 controls. The range of sample size ranged from 28 to 1311. There were 16 articles published in English between 1992 and 2018. Among the induced studies, 8 were from Asia, 6 from Europe, and 2 from American. Table 1 summarized the details characteristics and NOS score of the selected studies. (See Additional file 1).

### Statistical analysis

#### Endothelial cell density

**Fig. 2:**
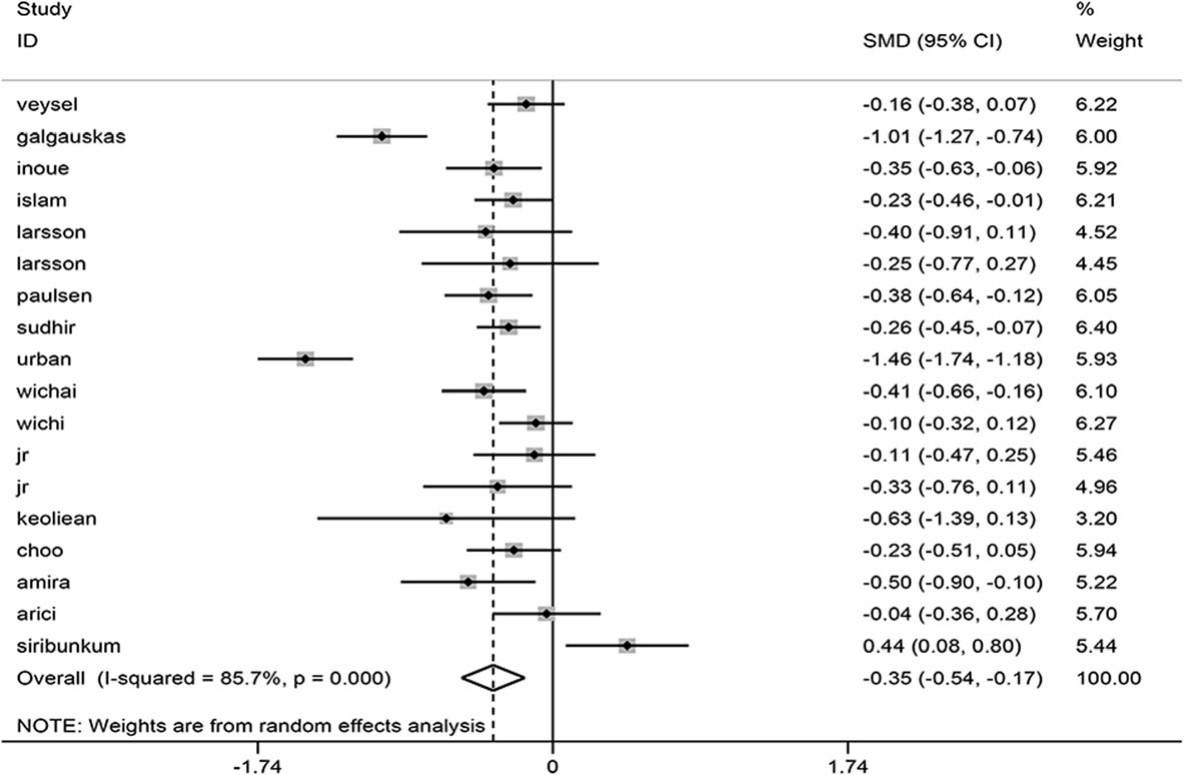
Forest plot of the effect of diabetes mellitus on ECD

A meta-analysis of corneal endothelial cell density included 18 studies involving 2887 cases and 1583 controls. Heterogeneity was detected in the study (I^2^ = 85.7%) so that a random-effects model was applied. According to the analytical results, the density of corneal endothelial cells in patients with diabetes was lower than in the healthy population, which was of statistical difference. (SMD = − 0.352, 95% CI -0.538, − 0.166, *P* = 0.000). (Fig. 2) The I^2^ value exceeded 50%, which evidences the heterogeneity among these studies. Subgroup analyses and meta-regression were conducted via the type of diabetes mellitus, study design, area, sample size, and the year of publication, while measurement tool was applied to identify the source of heterogeneity. It was found out that it is in type 2 diabetes (SMD = − 0.341, 95% CI -0.517, − 0.165) but not type 1 diabetes (SMD = − 0.685; 95% CI -1.390, 0.019), while type 1 and 2 diabetes (SMD = − 0.101; 95% CI -0.386, 0.185) exhibited a significant decline in ECD. When the measurement tools were identified, the significant change was observed in the non-contact specular microscope group (SMD = − 0.423; 95% CI − 0.642, − 0.203) but not in the contact specular microscope group (SMD = − 0.158; 95% CI − 0.465, 0.149). Considering the year of publication, the studies published after 2010 existed a significant difference (SMD = -0.450, 95% CI -0.710, − 0.191) but not before 2010(SMD = -0.191, 95% CI -0.401, 0.019). At study design subgroup, the significant result was obtained in cross-sectional studies rather than case-control studies. Besides, a subgroup analysis was conducted by study design and sample size. Nevertheless, the groups, including (Europe, Asian, American) and (size<200, size>200) were found to produce a significant result. The details about the subgroup analysis effect of diabetes mellitus on the ECD is presented. As indicated by the results of meta-regression, the type of diabetes mellitus might be contributing to heterogeneity. (*P* = 0.008) All results of the subgroup and meta-regression analyses for ECD are shown in Table 2.

**Table 2:** Subgroup analysis and meta-regression

Subgroup	No.of studies	Fixed-effects model		Meta-regression	Heterogeneity	
		SMD (95%)	*p*-value	*P* value	I^2^ (%)	*P*-value
All	18	−0.352(− 0.538, − 0.166)	0.000^*^		85.7%	0.000^*^
Diabetes mellitus type				0.008^*^		
Type1	4	−0.685(−1.390,0.019)	0.057		89.3%	0.000^*^
Type2	10	−0.341(− 0.517,-0.165)	0.000^*^		73.2%	0.000^*^
Type 1 and 2	4	−0.101(− 0.368,0.185)	0.491		79.9%	0.002^*^
area				0.823		
Europe	7	−0.501(−0.910,-0.092)	0.016^*^		92.6%	0.000^*^
Asia	8	−0.214(− 0.366,-0.061)	0.006^*^		63.0%	0.008^*^
American	3	−0.382(− 0.711,-0.053)	0.023^*^		0.0%	0.724
Study design				0.717		
Cross-sectional study	16	−0.391(−0.579,-0.203)	0.000^*^		84.9%	0.000^*^
Case-control study	2	−0.027(− 0.946,0.892)	0.95		91.5%	0.001^*^
Sample size				0.285		
< 200	10	−0.202(−0.379,-0.025)	0.026^*^		51.6%	0.029^*^
> 200	8	−0.494(− 0.793,-0.195)	0.001^*^		92.2%	0.000^*^
Publication year				0.842		
< 2010	8	−0.191(−0.401, 0.019)	0.075^*^		54.4%	0.032^*^
> 2010	10	−0.450(− 0.710,-0.191)	0.001^*^		90.6%	0.000^*^
Measurement tool				0.438		
Contact	12	−0.423(−0.642,-0.203)	0.000^*^		88.7%	0.000^*^
noncontact	6	−0.158(− 0.465,0.149)	0.312		61.5%	0.024^*^

* mean statistical difference

#### Mean cell area

**Fig. 3:**
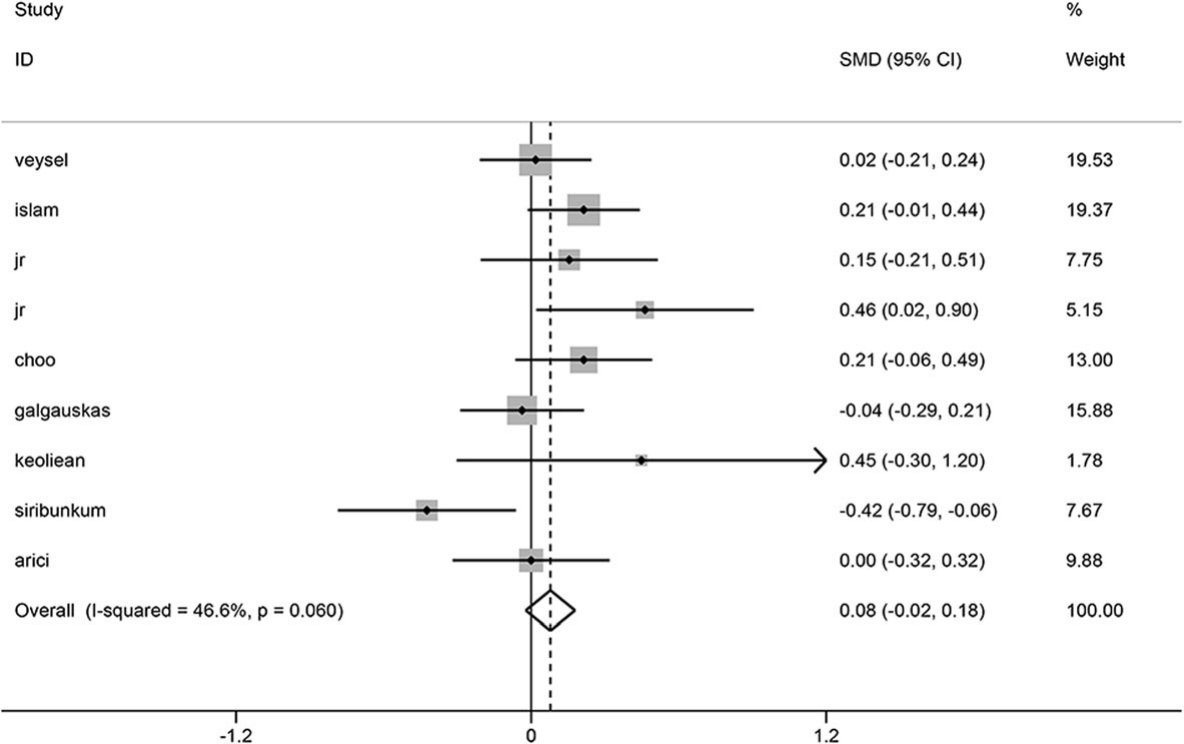
Forest plot of the impact of diabetes mellitus on MCA

The meta-analysis included a total of 9 studies involving 759 cases and 791 controls. No heterogeneity was detected in the study (I^2^ = 46.6%) so that a fixed effect model was applied. According to the analytical results, the Mean cell area of the patients with diabetes was not statistically different from that of the healthy population (SMD = 0.078, 95% CI -0.022, 0.178, *P* = 0.126) (Fig. 3).

#### Cell area variation coefficient

**Fig. 4:**
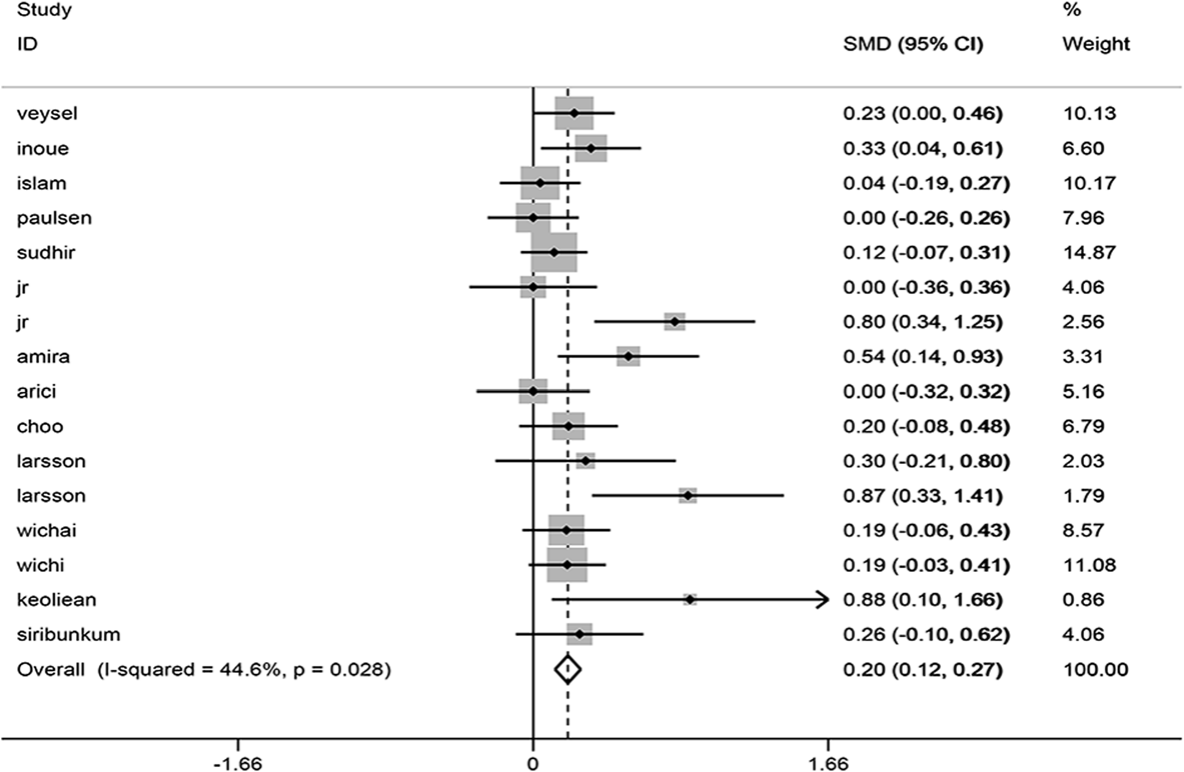
Forest plot of the impact of diabetes mellitus on CV

The meta-analysis included a total of 16 studies involving 2641 cases and 1339 controls. No heterogeneity was detected in the study (I^2^ = 44.6%), as a result of which a fixed effect model was applied. The analytical results showed that the coefficient of variation of corneal endothelial cells in patients with diabetes was higher than in the healthy population, with statistical differences observed (SMD = 0.195, 95% CI 0.123, 0.268, *P* = 0.000) (Fig. 4).

#### Hexagonal cell percentage

**Fig. 5:**
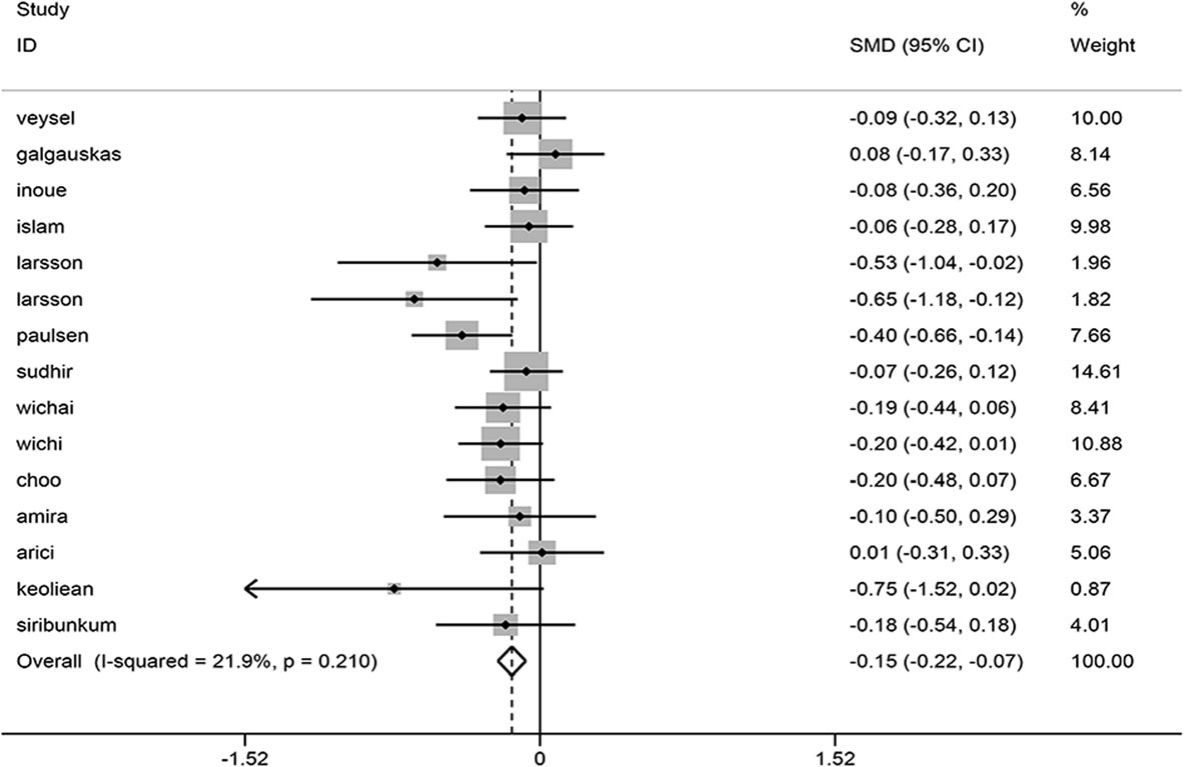
Forest plot of the impact of diabetes mellitus on HEX

Meta-analysis included a total of 16 studies involving 2664 cases and 1359 controls. There was no heterogeneity detected in the study (I^2^ = 21.9%) so that a fixed effect model was applied. The summary analysis revealed that the percentage of corneal endothelial hexagonal cells in patients with diabetes was lower than in the healthy population, which exhibited statistical differences. (SMD = − 0.145, 95% CI -0.217, − 0.074, *P* = 0.000) (Fig. 5).

### Sensitivity analysis

Sensitivity analysis, conducted by putting off one study at a time, indicated that none of the studies led to a significant change in the primary results, suggesting the stability and reliance of the findings. (See Additional file 2).

### Publication bias

Both Egger’ and Begg’s tests were conducted to evaluate the publication bias for four ECD, MCA, CV, and HEX. The results indicated a significant publication bias for studies, with combined CV (Begg’s test, *P* = 0.006; Egger’s test, *P* = 0.005) and merged combined HEX (Begg’s test, *P* = 0.113; Egger’s test, *P* = 0.024). Nevertheless, there was no apparent publication bias for combining ECD (Begg’s test, *P* = 0.363; Egger’s test, *P* = 0.669) and combining MCA (Begg’s test, *P* = 0.754; Egger’s test, *P* = 0.648) (Table 3).

**Table 3:** The result of publication bias estimation

	Egger’s test	Begg’s test
ECD	0.889	0.363
MCA	0.648	0.754
CV	0.005	0.006
HEX	0.024	0.113

## Discussion

Diabetes is known as a chronic disease associated with multiple systems, and any part of the eye is susceptible to its damage [47]. With the increasing number of intraocular surgery, diabetic corneal health has attracted increasing attention from ophthalmologists as it has the potential to influence postoperative recovery. Though there have been plenty of studies suggesting that diabetes damages corneal endothelium cell health, the quantitative evidence to prove this influence still is lacking. Therefore, a review was conducted of the published studies, and a meta-analysis was conducted in this study to make a more accurate estimate. According to the analysis, our results found that diabetes could damage corneal endothelium cell health. Cataract surgery led to more endothelium cells loss than healthy patients. So doctor should protect corneal endothelium cells during surgery.

The meta-analysis conducted by us combined 2 case-control studies and 16 cross-sectional studies, involving 2887 cases and 1583 controls. It was revealed that diabetes mellitus could reduce endothelial cell density (SMD = − 0.352, 95% CI -0.538, − 0.166). The I^2^ was found to be relatively high in the analysis of endothelial cell density, indicating obvious heterogeneity among these analyses. Therefore, a random effect model was chosen to minimize heterogeneity. In the subgroup analysis, the country-based subgroup analysis indicated a high level of heterogeneity in Asia and Europe. By contrast, the level of heterogeneity was zero in the American subgroup, suggesting that race might be a potential contributing factor for heterogeneity. According to meta-regression, the type of diabetes mellitus might be contributory to heterogeneity, which is speculated to have an association with the age of the patients involved in the study. Despite the presence of heterogeneity, our research revealed that diabetes could cause damage to the corneal endothelium. According to our observations, a previous meta-analysis could confirm our inference, but the results of this meta-analysis were inconsistent with our study. In the previous research, the focus was placed on the perioperative corneal endothelial research of diabetes and healthy patients with cataracts. The included literature was biased, and the data were insufficient. Therefore, this study was conducted as a supplement to it [48]. As revealed by studies, diabetes mellitus causes the AGEs to accumulate and excessive sorbitol accumulation in corneal endothelium, thus resulting in cell loss [49].

In addition, our results also revealed that diabetes mellitus could reduce the percentage of hexagonal cells (SMD = − 0.145, 95% CI -0.217, − 0.074), thus increasing the coefficient of variation of cell area (SMD = 0.195, 95% CI 0.123, 0.268). However, a similar incidence was shown when the mean cell area was analyzed (SMD = 0.078, 95% CI -0.022, 0.178). The I^2^ was found to be relatively low in the analysis of percentage of hexagonal cells, coefficient of variation of cell area and mean cell area. Therefore, a fixed-effect model was chosen. With corneal endothelium cells decreasing, the remaining corneal endothelium cells would be filled by the expansion and migration of neighboring cells. During the process, the shape of corneal endothelium cells loses regularity, thus causing an increase of CV and a decline of HEX. Meanwhile, diabetes inhibits the activity of Na^+^/K^+^-ATPase of the corneal endothelium, which plays a critical role in the maintenance of its structure [50].

Undeniably, the study is subject to some limitations. Firstly, our scope of the search was limited to the published articles, as a result of which those articles not published yet or grey literature with a possibility of meeting our inclusion criteria could have been discounted. Secondly, our study was heterogeneous. Besides, though subgroup analysis and meta-regression were conducted, its effect remained limited. The included articles were of low quality, and the number of included articles was insufficient. Otherwise, bias could have been detected by Begg’s test or Egger’s test. The language of included articles was restricted to English so that there might be another publication bias. Therefore, large samples, multiple centers, and high-quality research are deemed necessary for further research.

## Conclusion

In summary, it was found out that diabetes could reduce corneal endothelial cell density and the percentage of hexagonal cells but increase CV, indicating that diabetes causes the corneal endothelial cell to be unstable, which has detrimental effects on corneal endothelial cells.

## Supplementary Information

**Additional file 1.** Quality assessment.

**Additional file 2.** Sensitivity analysis.

## Data Availability

The datasets used and/or analyzed during the current study are contributed to by the corresponding author upon reasonable request.

## Abbreviations

ECD: Endothelial cell density
MCA: Mean cell area
CV: Cell area variation coefficient
HEX: Percentage of hexagonal cells
Nm: Not mention
CS: Cross-sectional
CC: Case-control
SMD: Standard Mean Difference
