# Supplementary material for: The effect of diabetes on corneal endothelium: a meta-analysis
Source: BMC Ophthalmol. 2021 Feb 10;21:78. doi: 10.1186/s12886-020-01785-3 (PMC7874671; doi:10.1186/s12886-020-01785-3)
Supplement: Supplementary file 1 — Additional file 1. Quality assessment. [file 12886_2020_1785_MOESM1_ESM.docx]

| Study | Selection |  |  |  | comparability |  | exposure |  |  | Score |
| --- | --- | --- | --- | --- | --- | --- | --- | --- | --- | --- |
| Veysel | a | a | c | a | a | b | b | a | b | 7 |
| Galgauskas | A | A | c | a | a | b | b | a | b | 7 |
| Inoue | a | a | c | a | a | b | e | a | b | 6 |
| Islam | a | a | b | a | a | b | b | a | b | 7 |
| Larsson | a | a | c | a | a | b | c | a | b | 6 |
| Paulsen | b | a | c | a | a | b | b | a | b | 6 |
| Sudhir | a | a | a | a | - | B | e | a | b | 6 |
| Urban | a | a | c | a | a | b | e | a | b | 6 |
| Wichai | c | a | c | a | a | b | e | a | b | 5 |
| Wichi | c | a | c | a | a | - | e | a | b | 4 |
| Jr | a | a | c | a | a | b | b | a | b | 7 |
| Keoliean | a | a | b | a | a | b | e | a | b | 6 |
| Choo | a | a | c | a | a | b | a | A | b | 7 |
| Amira | a | a | c | a | - | b | e | a | b | 5 |
| Siribunkum | c | a | c | a | a | b | e | a | b | 5 |
| Arici | a | A | c | a | a | b | e | a | b | 6 |
