# Supplementary material for: The effect of diabetes on corneal endothelium: a meta-analysis
Source: BMC Ophthalmol. 2021 Feb 10;21:78. doi: 10.1186/s12886-020-01785-3 (PMC7874671; doi:10.1186/s12886-020-01785-3)
Supplement: Supplementary file 2 — Additional file 2. Sensitivity analysis. [file 12886_2020_1785_MOESM2_ESM.docx]

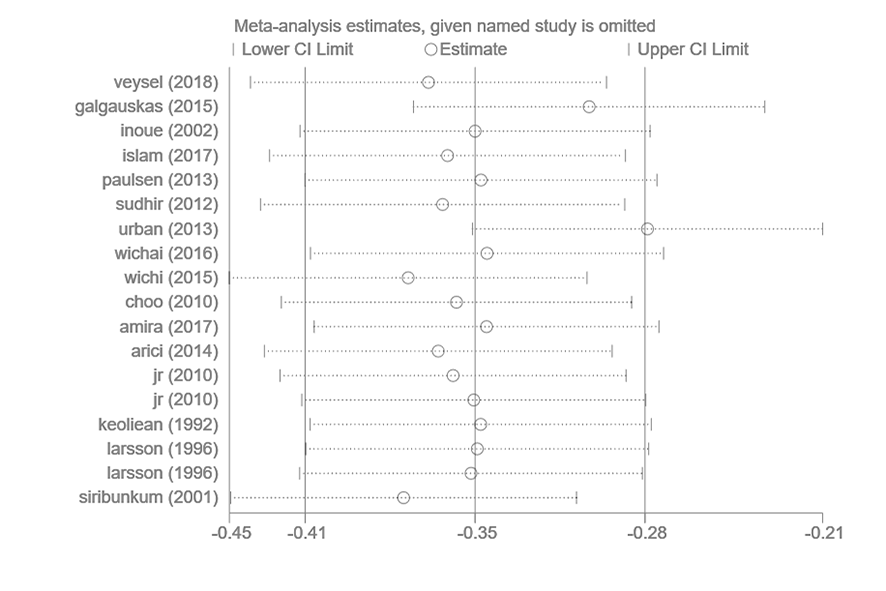


Sensitivity analysis of combining ECD


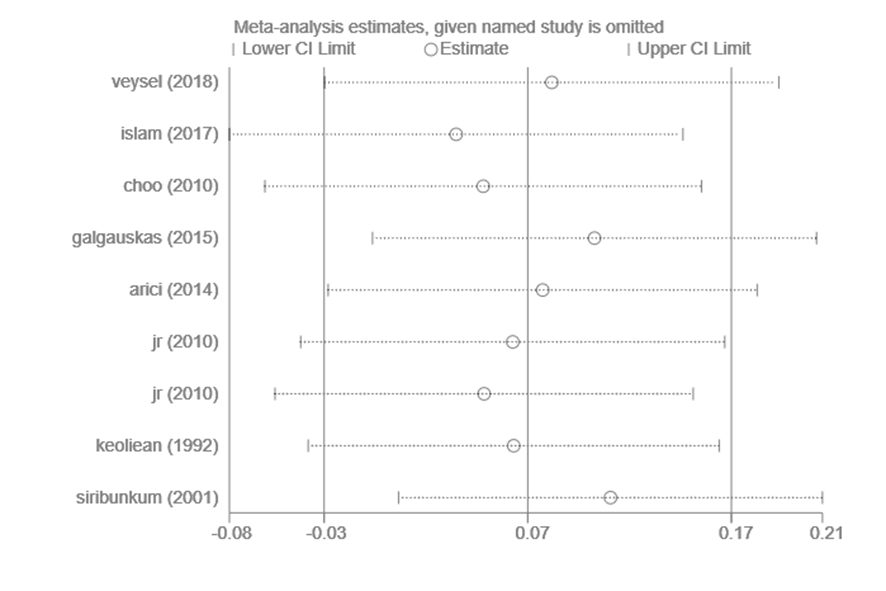


Sensitivity analysis of combining MCA


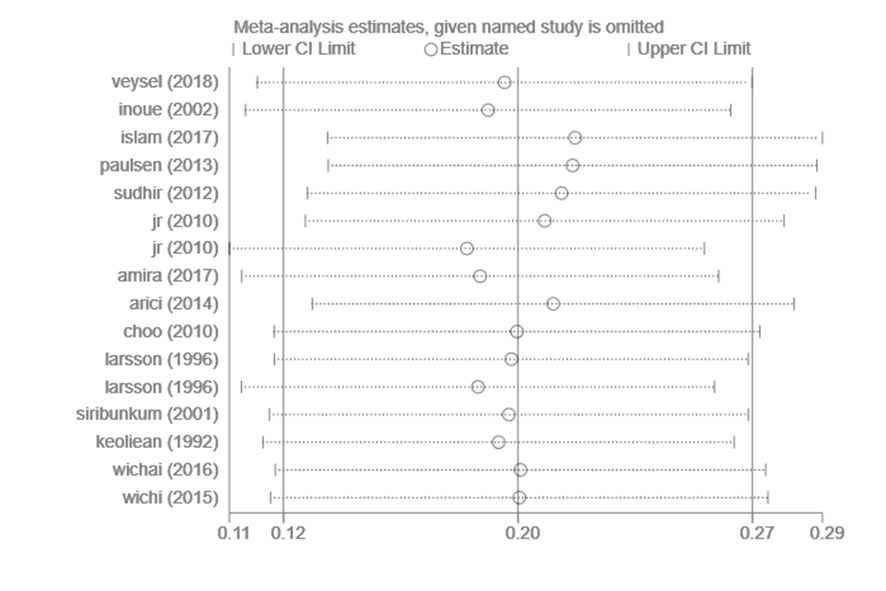


Sensitivity analysis of combining CV


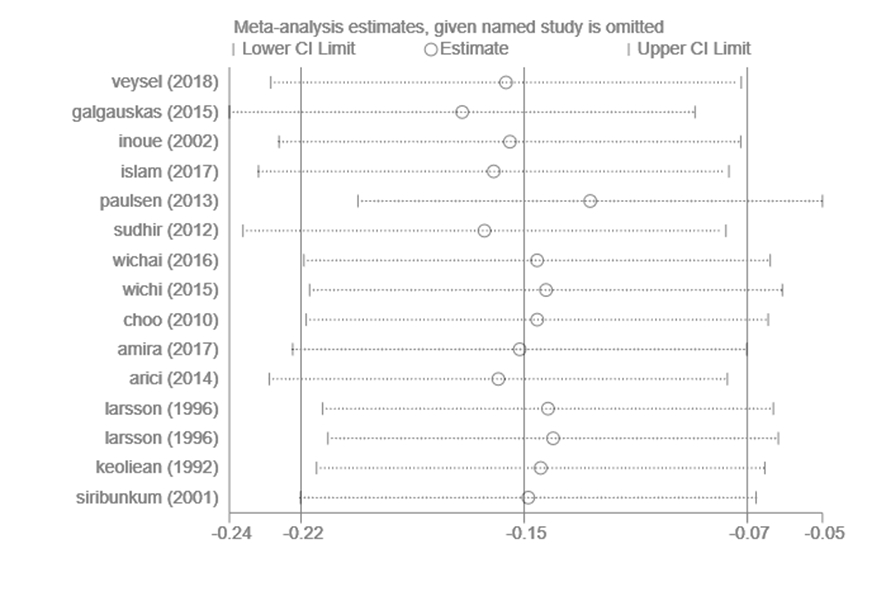


Sensitivity analysis of combining HEX
